# Unpacking COVID-19 Vaccine Hesitancy: A Network Analysis Perspective on Related Beliefs and Responses

**DOI:** 10.1007/s12529-025-10378-7

**Published:** 2025-06-16

**Authors:** Evangelos Karademas, Antonia Paschali

**Affiliations:** 1 University of Crete, Rethymno, Greece; 2 University of Nicosia, Nicosia, Cyprus; 3 National and Kapodistrian University of Athens, Athens, Greece

**Keywords:** Coronavirus, Pandemic, Vaccination, Vaccination hesitancy, Information processing

## Abstract

**Background:**

Although SARS-CoV-2 vaccination effectively prevents severe infection, many people hesitate to get vaccinated. Psychological factors contributing to vaccination hesitancy include beliefs about vaccine safety and effectiveness, perceived severity of infection, mistrust in authorities, misinformation and conspiracy beliefs, and pandemic-related distress and coping. This cross-sectional study, conducted in Greece, explored the relationships between vaccination and pandemic-related beliefs and responses and examined whether these relationships differed between vaccinated individuals and those hesitating or unwilling to vaccinate.

**Method:**

The study was conducted one year after the coronavirus vaccine was available for the entire population and the Omicron variant started to spread. The sample included 520 participants (358 females; mean age = 38.33 years). Network analysis was used to map the connections between the variables.

**Results:**

Findings revealed that people less concerned about infection severity and less confident in vaccine effectiveness were more likely to avoid vaccination. Notably, the network structure differed significantly between the two groups. Vaccinated participants exhibited a dense network of interconnected beliefs and responses, with vaccine safety, trust in authorities, worry regarding the pandemic or infection, and a positive outlook playing central roles. In contrast, the hesitant group displayed fewer connections, with vaccine safety and effectiveness beliefs linked primarily to trust in authorities and misinformation.

**Conclusions:**

These differences suggest that vaccinated individuals process pandemic and vaccine-related information more comprehensively, while hesitancy may stem from limited integration of related beliefs.

**Supplementary Information:**

The online version contains supplementary material available at https://doi.org/10.1007/s12529-025-10378-7.

## Introduction

Research has shown that vaccination against SARS-CoV-2 during the recent coronavirus pandemic was highly effective in preventing severe infection, hospitalization, ICU admission, and death [1, 2]. Individuals who were not vaccinated were at multiple risk for all of the above compared to those vaccinated [3]. However, one of the problems highlighted by the pandemic was that a significant part of the population could not include or process all available information regarding vaccination against the virus so as to reach an informed decision. Hence, many worldwide hesitated to get the vaccine [4].

The global prevalence of coronavirus vaccination hesitancy was estimated at about 25% [5], but significant variance existed across countries, time, and population. Female gender, younger age, lower socioeconomic status, and lower annual seasonal influenza vaccination level were related to higher hesitancy [5–10]. Health status (e.g., severe or chronic illness), healthcare provider recommendations, and not relying on social media for relevant information were related to a higher possibility of uptaking the vaccine [10–12].

Moreover, several cognitive-emotional factors were correlated to a higher risk of vaccination hesitancy. The most significant of them were (a) considering the vaccines against the coronavirus as less safe, (b) having concerns about the effectiveness of the vaccine, (c) mistrust in governmental or scientific institutions and dislike of forced measures, (d) less adherence to the pandemic-related protective measures (e.g., wearing a mask in crowded places), and (e) doubts regarding the severity or probability of a coronavirus infection [5–7, 11, 13, 14]. Remarkably, these same reasons were reported in many studies from different countries and populations [14, 15]. The same reasons also seemed to concern health professionals unwilling to be vaccinated [16, 17].

Lower levels of COVID-19-related distress [18], decreased fear of COVID-19 [8], and misinformation or conspiracy beliefs (e.g., regarding the role of pharmaceutical companies or 5G networks in the outburst of the pandemic) have also been associated with higher hesitancy [19–22]. However, the impact of conspiracy beliefs probably faded with time [23], while there is some evidence that conspiracy theories were used only to rationalize already existing vaccination hesitancy [24].

Interestingly, the studies that examined the factors associated with coronavirus vaccination hesitancy primarily focused on potential mean differences between those who got (or said they would get) the vaccine and those who were unwilling/hesitating to be vaccinated. The relationships between these factors were not examined substantively, although the examination of these relationships may reveal deeper dynamics and processes about how vaccination-related factors interact.

This study aimed to explore how major determinants and correlates of vaccination hesitancy, as highlighted by the relevant previous research, are interrelated (1st research question). Specifically, perceptions of the coronavirus vaccine (e.g., regarding its safety and effectiveness), mistrust in official institutions, conspiracy beliefs, previous protective behaviours, infection-related perceptions (e.g., regarding its severity), as well as pandemic-related distress and ways of coping with it were included in the study as representing different aspects of the pandemic experience.

At the same time, the study aimed to examine whether the strength of these relationships differs among persons vaccinated and persons hesitating/unwilling to be vaccinated (2nd research question). The potential differences between these two groups are likely due to divergent ways of processing relevant information, and this may be reflected in the overall structure of the links between the vaccination-related variables.

The study aimed to map the complex direct and indirect relationships between various variables relevant to vaccination hesitancy as a first step towards understanding their overall structure rather than to examine specific hypotheses about particular associations. Of course, we expected significant links between certain variables. For example, we anticipated significant associations between trust in official governmental or scientific institutions and beliefs about the safety and efficacy of vaccines [6, 21] or between coronavirus protection behaviours, covid-related distress, and beliefs about the infection and the vaccine [4, 6–8, 14, 18]. We also expected some of these relationships to differ between groups. For example, mistrust and conspiracy beliefs may be more strongly related to the beliefs about the safety and efficacy of vaccines in the group of those who hesitated to be vaccinated [23, 25].

Still, the study’s primary goal was to investigate the general patterns of the associations between the abovementioned variables, as well as the possible differences between vaccinated and hesitant persons. To this end, we used network analysis. Network analysis is a powerful alternative to classical statistical methods. It focuses on the relationships and interactions between variables or entities rather than treating them as individual units. Unlike traditional statistical analyses that often assume independence between observations, network analysis captures the structure and strength of connections within a system. By visualizing and quantifying relational data, network analysis provides a deeper insight into the dynamics of interconnected systems [26]. By using network analysis, it will be possible to gain a broad understanding of how the network of responses to the pandemic and vaccination possibly differs between groups. This may contribute to a better understanding of how the general population responds to the need for mass vaccination due to a public health emergency and, in turn, to practical suggestions for developing more effective vaccination promotion methods in similar conditions.

## Method

### Participants and Procedure

This cross-sectional study was conducted in Greece from November 2021 to January 2022, one year after the coronavirus vaccine became available for the entire population. During that period, the country was navigating the challenges of the fourth wave of the pandemic (the beginning of the Omicron variant spreading; a version of the coronavirus that spreads very quickly but is usually associated with milder disease than previous variants; https://www.emro.who.int/health-topics/corona-virus/omicron-voc-questions-and-answers.html) and the majority of the population had been vaccinated (https://www.data.gov.gr/datasets/mdg_emvolio/). The study was conducted online through a survey portal and was approved by the Ethic Committees of the University of Crete (No 144/04.11.2021) and of the Faculty of Nursing, National & Kapodistrian University of Athens (No 380/19.11.2021). Participants were invited to complete the form with the link shared on social media platforms (Facebook and X/Twitter) and through local announcements (e.g., local news sites). Inclusion criteria were being an adult, understanding the study protocol and providing informed consent.

The sample consisted of 520 individuals (162 males and 358 females; mean age = 38.33 years, SD = 14.78). Of the participants, 14.8% had finished only the mandatory 9-year education, 12.3% were university students, and 72.9% were holders of a higher education degree; 31.3% reported a high income, whereas the remainder reported a low to medium income; 71.2% were living with their family or partner; 19% reported a chronic disease (e.g., diabetes, cardiovascular disease).

Over 60% reported that they or a very close person/family member was infected by the coronavirus. Finally, 85.96% reported that they had received one of the available anti-coronavirus vaccines, whereas 14.04% reported that they were not willing to get the vaccine at all or had received only one of the multiple doses required but did not plan to complete the vaccination scheme.

### Measures

**Infection-related beliefs**. (a) Infection representations. A single-item approach, based on the Brief Illness Perception Questionnaire [27], was employed to assess representations about coronavirus infection. Specifically, participants were asked to reply to four short questions about their relevant experience using a Likert-type scale ranging from 1 (not at all) to 10 (very much/very well). The questions were the following: “how much do you think the life of those infected by the coronavirus is affected” (infection consequences); “how much do you think existing treatments can help the infected patients” (treatment control); “how severe do you think the symptoms of the COVID-19 are for those infected” (infection severity); “how concerned are you about the possibility of getting infected (or reinfected) by the coronavirus” (worry about the possibility to get infected). (b) Trust in institutions. To assess trust in governmental and health system-related institutions or agencies, participants were asked to answer four questions developed for this study (e.g., how much do you trust the instructions and decisions of the government and state agencies on pandemic issues; how much confidence do you have in the instructions and decisions of the special scientific/health system committees on pandemic issues; Cronbach *a* = 0.91) using a Likert-type scale ranging from 1 (not at all) to 5 (very much).

**Vaccine-related beliefs**. (a) Vaccine safety. To assess the perceived safety of the available vaccines, three questions were used (i.e., In general, the coronavirus vaccines are… safe/useful/dangerous; Cronbach *a* = 0.87) with a Likert-type response scale ranging from 1 (totally disagree) to 5 (totally agree). (b) Vaccine effectiveness was assessed with three questions (i.e., In general, the coronavirus vaccines are… effective/suitable for all/not effective in the long run; Cronbach* a* = 0.78) with the same response scale. (c) Vaccine-related conspiracy beliefs were assessed with four questions (e.g., In general, the coronavirus vaccines are… a manner of control by the State or the big companies; …based on false evidence; Cronbach *a* = 0.80). All questions concerning vaccine-related beliefs were developed for this study.

**Pandemic-related responses**. (a) Six protective behaviours against infection were assessed (e.g., how often do you… wash your hands or use antiseptic; …use a mask in crowded spaces; …keep the appropriate physical distance from others). Respondents used a 5-point Likert-type scale ranging from 1 (very rarely/never) to 5 (always). The overall score was used to indicate the general degree of adherence to protection measures against infection (Cronbach *a* = 0.81). (b) Coping with the pandemic. The Ways of Coping scale [28], as adapted in Greek [29], was used to assess coping behaviours concerning the pandemic-related demands (e.g., restrictions in social life and work) and worry. Participants indicated how much they used each coping behaviour during the past few weeks on a Likert-type scale from 0 (not used) to 3 (used a great deal). An exploratory factor analysis (Kaiser–Meyer–Olkin Test = 0.83; Bartlett’s test of Sphericity *p* < 0.001) using maximum likelihood extraction with a varimax rotation revealed five factors which explained 58.66% of the total variance: (i) positive coping, which refers to a combination of problem-solving and positive reappraisal (11 items, e.g., tried to find solutions to the situation and its consequences; changed something so things would turn out all right; Cronbach *a* = 0.84); (ii) seeking social support (7 items, e.g., relied on the support and help of those around me; Cronbach *a* = 0.81); (iii) a “wait-and-see” approach (7 items, e.g., was waiting to see how things would go; Cronbach *a* = 0.72); (iv) wishful thinking (5 items, e.g., wished I could change what's happening; Cronbach *a* = 0.73); (v) religious coping (3 items, e.g., found comfort in my faith in God; Cronbach *a* = 0.80). (c) COVID-19 related perceived stress was assessed with the Perceived Stress Scale [30], as adapted for the pandemic by Campo-Arias et al. [31]. It includes 10 items (e.g., I felt nervous or stressed about the pandemic). Participants were asked to reply using a Likert-type scale ranging from 0 (never) to 4 (always), based on their experience over the past few weeks. Higher total scores correspond to more COVID-19 related stress (Cronbach *a *= 0.83).

### Analyses

Chi-square tests were used to examine the association of the vaccination status (i.e., vaccinated vs. hesitating to complete vaccination) with the sociodemographic variables. A MANCOVA, with vaccine and COVID-19 related beliefs and responses as the dependent variables and the vaccination status as the independent variable, was used to identify their relationships after controlling for the sociodemographic variables. A significance level of 0.01 (for two-sided statistical tests) was employed.

We used network analysis to examine the overall structure of the relations between vaccine and COVID-19 related beliefs and responses in the two groups of vaccination status. Network analysis is a major methodological means for examining complex relationships (called ‘edges’) between interconnected variables (called ‘nodes’), identifying patterns of associations, and visualizing these complex links [31]. Network analysis allows the concurrent examination of all possible reciprocal relationships between the nodes [32]. For example, it may allow us to identify direct relationships (edges) between variables (nodes), as well as indirect relations (i.e., how a node is associated with another through other nodes). Each edge has a “weight”, which shows how strong the connection (edge) between two nodes is, after accounting for all other variables in the network.

Network analysis was performed using JASP [33], an open-source statistics program based on R. The network was built on partial correlations (i.e., the association between two nodes is controlled for the impact of all others) using the Extended Bayesian Information Criterion Graphical Least Absolute Shrinkage and Selection Operator (EBICglasso) which retains only meaningful associations. The γ value, which determines how much sparser models (i.e., more parsimonious with fewer edges) will be preferred, was set to 0.50. Also, the Fruchterman-Reingold algorithm was used to organise the network based on the strength of the connections between the nodes.

Three centrality measures were used to identify the importance of each node [26]: (a) betweenness, which reflects how important a node is in connecting other nodes; b) closeness, which reflects a node’s direct and indirect relationship to all other nodes and indicates how quickly this node may affect and be affected by changes in the network; (c) node strength, which is the most important centrality measure and indicates how strongly a node is connected to other nodes. However, centrality indices are sensitive to sample size and variability and, therefore, to examine node strength stability (i.e., whether the order of the nodes’ strength remains the same after re-estimating the network with fewer cases or nodes), we used the correlation stability coefficient (CS-coefficient). The coefficient should not be below 0.25 and preferably over 0.50 [34]. Also, to examine potential differences in worthy of note edges across the two groups of vaccination status (i.e., whether the links between variables actually differ between vaccinated and hesitating participants), we used the R package Net Comparison Test (NCT) [35]. Finally, sparsity value refers to the number of edges in a network. A value closer to 1 indicates a sparser network with few non-zero edges.

## Results

### Preliminary Results

The means and standard deviations of all vaccine and COVID-19 related beliefs and responses for the entire sample and across vaccination status are presented in Table 1. Vaccination status was associated with education level (high education degree vs. no degree) and income (high vs. low), chi-square > 8.50, *p* < 0.01. Concerning education level, 22% of the non-holders of a high-education degree hesitated to be vaccinated vs. 11.1% of holders. Concerning income, 20.2% of the lower-income participants hesitated to be vaccinated vs. 10.8% of those with high income. There was no statistically significant association with gender, personal status (living with someone vs. living alone), and chronic illness (having a chronic illness or not), chi-squares < 3.75, *p* > 0.01. Also, hesitating to be vaccinated participants were younger (34.21 years vs. 38.99), *F*(1, 518) = 6.67, *p* < 0.01.

**Table 1:** Means and standard deviations of vaccine and COVID-19 related beliefs and responses for the entire sample and across vaccination status

	Entire sample (*N* = 520)	Vaccinated (*N* = 447)	Hesitating (*N* = 73)
**Infection-related beliefs**			
Consequences	8.37 (1.73)	8.53 (1.59)	7.40 (2.19)*
Treatment control	5.87 (1.91)	5.91 (1.91)	5.60 (1.98)
Severity	7.39 (1.69)	7.55 (1.64)	6.49 (1.71)*
Possibility	6.85 (2.52)	7.10 (2.34)	5.29 (3.04)*
Trust in the government/health system	4.38 (1.55)	4.55 (1.47)	3.32 (1.63)*
**Vaccine-related beliefs**			
Safety	4.21 (.82)	4.36 (.66)	3.29 (1.09)*
Effectiveness	3.57 (.98)	3.73 (.86)	2.56 (1.08)*
Conspiracy beliefs	1.73 (.87)	1.58 (.72)	2.62 (1.16)*
**Pandemic-related responses**			
Protective behaviours	3.90 (.85)	3.97 (.80)	3.47 (.99)*
Positive coping	2.68 (.64)	2.69 (.63)	2.62 (.69)
Social support	2.50 (.70)	2.50 (.69)	2.53 (.73)
‘Wait-and-see’ coping	2.34 (.66)	2.32 (.67)	2.47 (.57)
Wishful thinking	2.59 (.77)	2.57 (.77)	2.71 (.75)
Religious coping	1.61 (.80)	1.55 (.74)	1.97 (1.02)*
COVID-19 related stress	2.78 (.74)	2.76 (.74)	2.91 (.73)

^*^p <.001, after controlling for sociodemographic variables

According to the results of the MANCOVA, after controlling for all sociodemographic variables, vaccination status was associated with several vaccine and COVID-19 related beliefs and responses, Wilks *λ* = 0.76, *F*(15, 498) = 10.75, *p* < 0.001, partial *η*^2^ = 0.25. Specifically, vaccinated participants reported higher levels of infection consequences, severity and possibility, trust in governmental and health system institutions, vaccine-related safety and effectiveness, and protective behaviours, as well as lower levels of conspiracy beliefs and religious coping, *F*s > 16.60, *p* < 0.001, partial *η*^2^s = 0.03–0.19.

### Structure of Correlations Across Vaccination Status

**Fig. 1:**
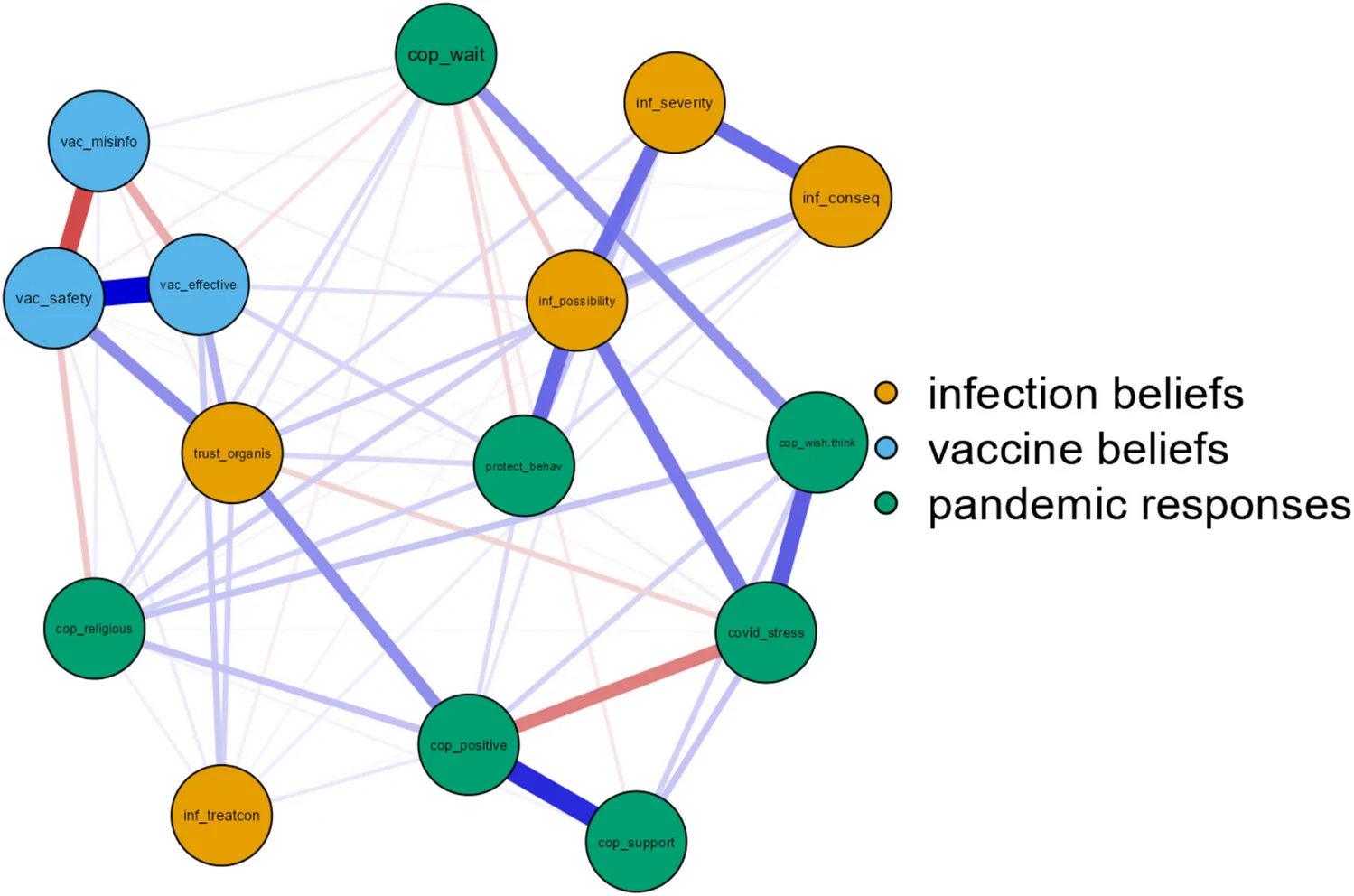
Visualisation of the network of the vaccine and pandemic-related beliefs and responses: vaccinated participants. *Note*. Inf = infection related; conseq = consequences; treatcon = treatment control; vac = vaccine related; misinfo = misinformation/conspiracy theories; cop = coping; protect_behav = protective behaviours. Blue and red lines represent positive and negative associations, respectively. Thicker lines correspond to stronger associations

**Fig. 2:**
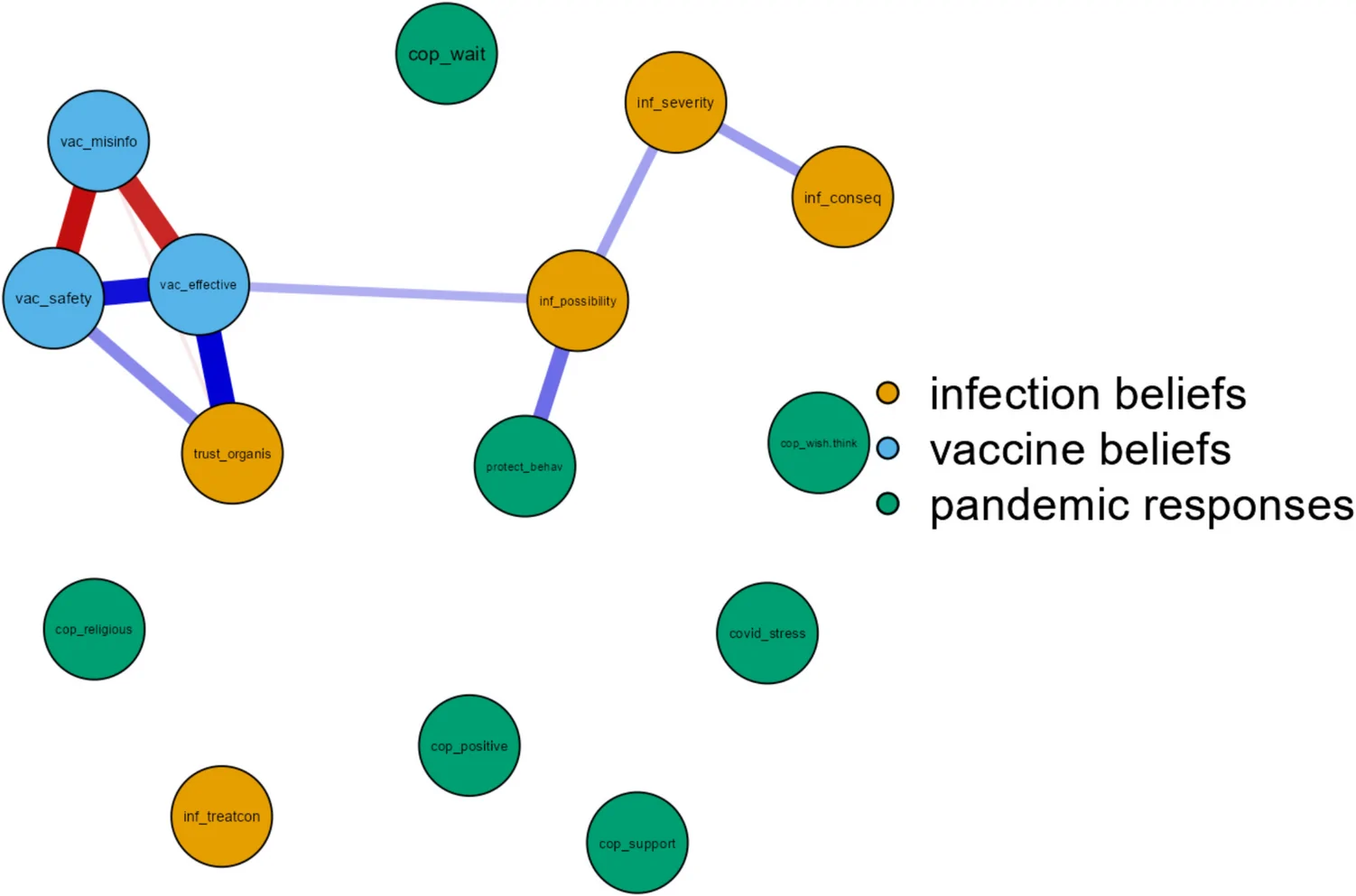
Visualisation of the network of the vaccine and pandemic-related beliefs and responses: hesitating to be vaccinated participants. *Note*. Inf = infection related; conseq = consequences; treatcon = treatment control; vac = vaccine related; misinfo = misinformation/conspiracy theories; cop = coping; protect_behav = protective behaviours. Blue and red lines represent positive and negative associations, respectively. Thicker lines correspond to stronger associations

**Fig. 3:**
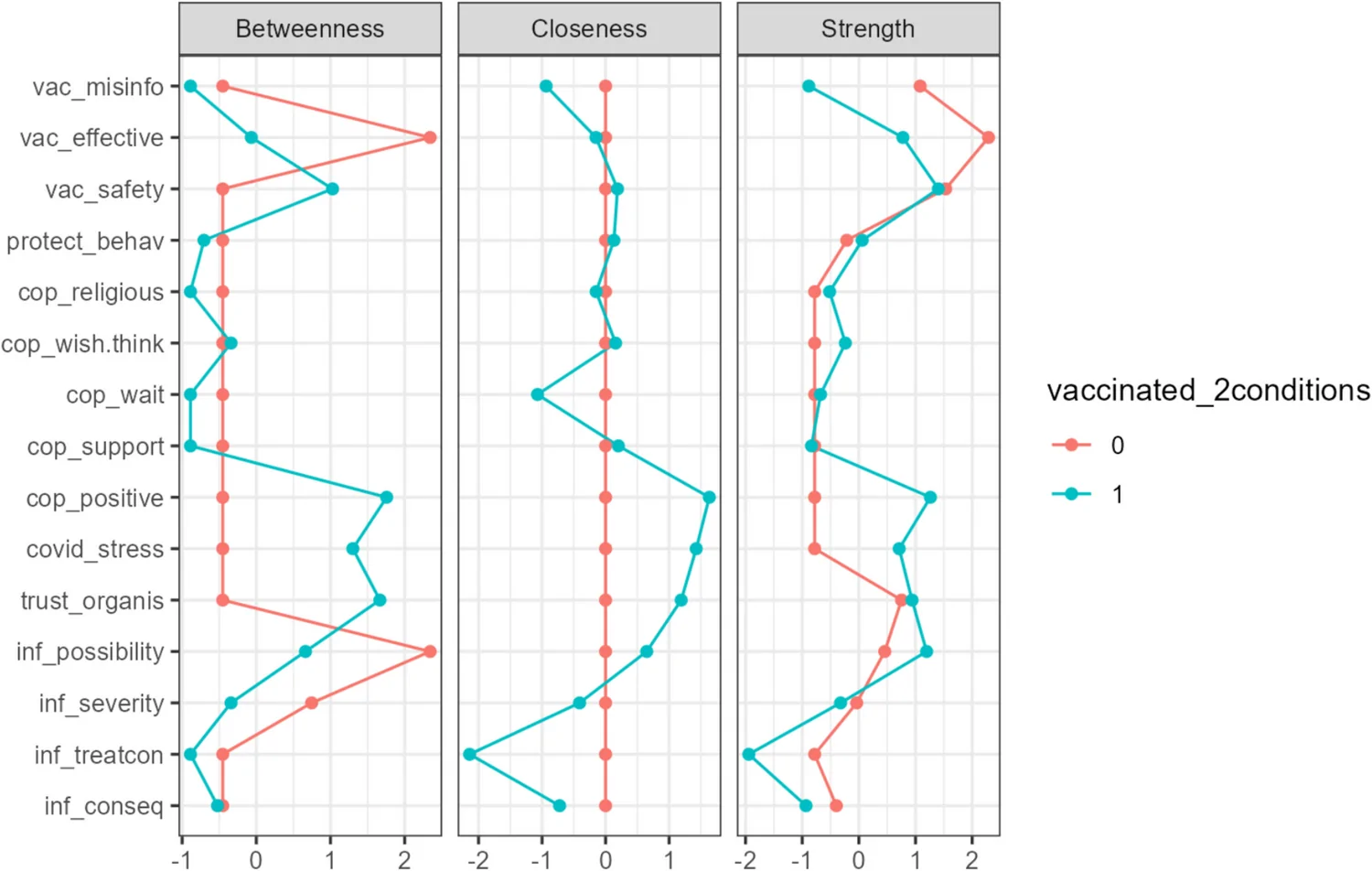
Centrality indices for the network of the vaccine and pandemic-related beliefs and responses. *Note*. Vaccination status: 0 = hesitating to be vaccinated participants; 1 = vaccinated participants. Inf = infection related; conseq = consequences; treatcon = treatment control; vac = vaccine related; misinfo = misinformation/conspiracy theories; cop = coping; protect_behav = protective behaviours

Figures 1 and 2 present the network structure for the two groups of vaccination status, and Fig. 3 shows the centrality measures. Supplementary Figs. 1 to 3 (please see Supplementary File) show the 95% confidence intervals (CI) of the network edge weights and the stability of centrality measures (number of bootstraps = 2500; non-parametric bootstrapping). Regarding the edge weights, the confidence intervals are rather broad, and thus the order of edge estimates should be interpreted with caution [34]. Still, several edges in both samples were reliably the strongest as their bootstrapped CIs do not overlap with the CIs of the other edges (see Supplementary Figs. 1 and 2).

Concerning vaccinated participants (see Fig. 1), vaccine beliefs (i.e., regarding their safety, effectiveness, and relevant conspiracy beliefs) were closely interconnected (edge weights = −0.15 to 0.47; safety and effectiveness were negatively related to conspiracy beliefs). Several variables were associated with vaccine beliefs, with trust in governmental/health system institutions showing a stronger association with vaccine safety (edge weight = 0.21) and a weaker association with vaccine effectiveness (edge weight = 0.14), as indicated by the bootstrapped CIs. Protective behaviours, worry about infection possibility, infection treatment control, and religious coping were also weaklier linked with vaccine beliefs (edge weights = 0.07 to 0.11). Moreover, trust in institutions was strongly associated with positive coping (edge weight = 0.21), and weaklier with infection treatment control, infection consequences, and protective behaviours (edge weights = 0.07 to 0.13). Links were also found between positive coping and social support, worry about infection possibility and protective behaviours as well as infection severity, infection consequences and infection severity, wishful thinking and covid-related stress as well as worry about infection possibility (edge weights = 0.13 to 0.30).

Besides direct relations, several indirect ones were noticed. For example, social support was connected to trust in institutions through positive coping, and COVID-19 related stress was connected to protective behaviours through worry about infection possibility (Fig. 1). Overall, the network structure was dense, demonstrating many node connections (63 non-zero edges out of 105; sparsity = 0.400). As shown in Fig. 3, the nodes with the highest strength values were vaccine safety, positive coping, and worry about infection possibility. The nodes with the highest betweenness and closeness values were positive coping, trust in institutions, and COVID-19 related stress. The CS-coefficient was 0.36, suggesting a moderate strength stability.

On the other hand, the network structure concerning participants hesitating to be vaccinated was very sparse, with only a few non-zero edges (10 out of 105; sparsity = 0.905). As in vaccinated participants, vaccine beliefs were closely interconnected (edge weights = −0.23 to 0.25). Trust in institutions was strongly associated with vaccine effectiveness (edge weight = 0.27) and weaklier to vaccine safety (edge weight = 0.13; see Fig. 2). Also, vaccine effectiveness was associated with worry about infection possibility, which in turn was associated with infection severity and protective behaviours, and infection severity was linked with infection consequences (edge weights = 0.09 to 0.16). However, these links were weaker, as indicated by the bootstrapped CIs.

Concerning the non-zero edges in the hesitating participants, there were no significant differences with the vaccinated participants, except for the edge between trust in the government/health system and vaccine effectiveness, which was stronger in the hesitating participants (*p* < 0.05), as indicated by the Network Comparison Test. Finally, as shown in Fig. 3, the nodes with the highest strength values in the group of hesitating participants were vaccine safety and vaccine effectiveness. The nodes with the highest betweenness values were vaccine effectiveness and worry about infection possibility. The CS-coefficient was 0.29, also suggesting a moderate strength stability.

## Discussion

In this study, we aimed to examine the structure of the relationships between various variables relevant to COVID-19 vaccination hesitancy in two groups: fully vaccinated vs. persons hesitant to be vaccinated or complete the vaccination scheme. Analysing the possible differences between these two groups may contribute to a better understanding of the general population’s responses to the need for mass vaccination and the corresponding processes. The study provided interesting results.

As highlighted in several previous studies (e.g., [5–7]), individuals hesitating to get vaccinated tended to be younger, with lower income and educational level. They also appeared less concerned about the possibility of a coronavirus infection and its severity and consequences. Additionally, they were less convinced about the vaccine’s safety, effectiveness and purpose, exhibited lower trust in governmental and health institutions, and were more likely to rely on religious coping, as per existing research [7, 11, 15, 20]. Overall, it seems that people who are less concerned about the health impact of a potential infection and less convinced about the protective value of the existing vaccines are more likely to avoid vaccination.

The most important finding is the striking difference in the network structures of the two groups of participants. The number of edges (i.e., connections between variables) in vaccinated participants was multiple of the number of edges in participants hesitating to be vaccinated.

Concerning the vaccinated participants, beliefs regarding vaccine safety, effectiveness, related conspiracy theories, and trust in governmental and health system institutions were, as expected, closely related [6, 21] – a quartet of strongly interconnected variables. In turn, they were linked to other factors, such as protective behaviours against infection, worry about infection possibility, perception of infection severity, and positive coping. Previous research has also demonstrated several of these relationships [4, 6, 14, 18]. However, the findings of this study further revealed a dense network of direct and indirect relationships among important vaccine and pandemic-related responses. A possible explanation of this finding might be that there is a broad feedback mechanism that probably allows for a freer circulation of"information"and facilitates crucial responses (like vaccine-related perceptions and use of protective behaviours) to be informed and shaped by a constellation of other attitudes and beliefs. Moreover, it possibly allows important decisions (e.g., regarding vaccination) to be determined by several parameters and after considering different aspects of the entire experience with the pandemic. Of course, this is a possibility that warrants further research.

Three variables displayed the larger number of links to other variables (node strength): vaccine safety, positive coping, and worry regarding infection possibility. In addition, positive coping, trust in institutions, and COVID-19 related stress showed the highest betweenness and closeness values. That is, these variables were more important in connecting others and displayed the highest sensitivity to changes in the network (i.e., they swiftly affect and are affected by changes in the other variables). It seems that a positive approach to the situation, belief in vaccine suitability, trust in the official bodies responsible for dealing with the situation, and worry about the pandemic or a possible infection have a crucial role in the network of pandemic-related beliefs and responses. These variables may serve as major intervention points to promote vaccination in the general population.

On the other hand, only a few connections between the variables were found in those hesitating to uptake the vaccine or complete the vaccination scheme. Interestingly, the two factors that are critical for vaccination behaviour (i.e., beliefs about vaccine safety and effectiveness [7, 14]) were strongly linked only to related conspiracy theories and (mis)trust in governmental and health system institutions (and, weaklier, to infection possibility). Individuals hesitant to receive the vaccine may have formed their opinions and made vaccination-related decisions based on a limited set of key factors, such as their level of trust in official institutions and exposure to conspiracy theories, potentially overlooking other important parameters systematically. Besides these, only a few other weaker links were found: protective behaviours were associated with worry about infection possibility, which was also associated with infection severity and consequences. Important factors, such as COVID-19 related stress and positive coping, were unrelated to other variables. In other words, the personal pandemic and vaccine-related beliefs and responses seemed to remain independent from one another and not form a feedback network. Several reasons may underlie this finding, such as complex pathways through variables not assessed here or the impact of broader social, personality and other factors special to this group of participants. Overall, although more research is needed to explain this intriguing finding, the observed differences between the two networks, in terms of node centrality and number and type of non-zero edges, reveal significant differences in the ways that the two groups of participants structured their Covid- and vaccine-related responses.

The study has limitations and the findings should be considered in light of them. As this is a correlational study, the direction of the relationships remains unclear. The number of participants (especially in the group of people hesitating to be vaccinated) was modest for network analysis. This might be the reason underlying the differences between the two groups of participants and the fact that the stability of strength centrality in both groups was moderate, indicating that our centrality findings are reasonably robust, though not highly stable. Further research with larger samples is needed to examine the differences between vaccinated and hesitant-to-vaccinate people. Almost two-thirds of the participants were females, and the majority were rather well-educated. In addition, sampling biases, which are often involved in online studies (e.g., older adults are less likely to participate), may also be present here. Although many variables were examined, several others that may be important for vaccination hesitancy, like personal decision-making style, social influence, and healthcare-related factors, were not assessed. The study was conducted about a year after the coronavirus vaccine became available for the country’s general population. Thus, although the study reflects a time when the vaccine was readily available to everyone, it does not reflect previous or later conditions regarding the COVID-19 pandemic.

In conclusion, despite the limitations, the findings of this study are significant as they indicate that the key to understanding vaccination hesitancy may lie in deciphering how information flows between the factors that people mostly take into account when making vaccination-related decisions. Further research is needed to examine this issue. Moreover, the findings underline the importance of positive coping, trust in institutions, and worry regarding the pandemic or a possible infection. In order to achieve changes in significant responses (like protective behaviours and vaccine-related beliefs), these factors might be considered as the focal points of potential interventions. However, at the same time, the findings indicate that interventions or health policies especially targeting individuals hesitant to be vaccinated should be differentiated from those designed for the less hesitant individuals. It is crucial to focus more on vaccine-hesitant people and use information gained by relevant research in order to develop appropriate interventions that may help them increase the integration of scientific evidence (i.e., that vaccines are generally effective and safe) in their vaccination-related decisions. Based on the findings of this study, these interventions should rather focus on the variables that seem to be central in their vaccination-related behaviour. Namely, trusting official institutions to make the best possible decisions and efficiently and promptly addressing misinformation and conspiracy theories about vaccines or vaccination policies. Although the number of relevant efforts is increasing, they still face several limitations [36–38]. Therefore, it is essential to develop effective and easy-to-use intervention strategies to challenge these attitudes.

## Supplementary Information

Below is the link to the electronic supplementary material.

Supplementary file1 (DOCX 183 KB)

## Data Availability

The fully anonymized data that support the findings are available on request from the authors. The data is not publicly available because of privacy and ethical restrictions.
