# Supplementary material for: Unpacking COVID-19 Vaccine Hesitancy: A Network Analysis Perspective on Related Beliefs and Responses
Source: Int J Behav Med. 2025 Jun 16;33(4):621–9. doi: 10.1007/s12529-025-10378-7 (PMC13624034; doi:10.1007/s12529-025-10378-7)
Supplement: Supplementary file 1 [file 12529_2025_10378_MOESM1_ESM.docx]

**Supplementary File**

Supplementary Figure 1. *The accuracy of the edge weight estimates (red line) and the 95% bootstrapped confidence intervals (grey area; bootstraps number = 2500) for the vaccinated participants. The names of the edges have been removed for clarity reasons.*

Supplementary Figure 2. *The accuracy of the edge weight estimates (red line) and the 95% bootstrapped confidence intervals (grey area; bootstraps number = 2500) for the participants hesitating to be vaccinated. The names of the edges have been removed for clarity reasons.*

|  |  |
| --- | --- |

Supplementary Figure 3. *The stability of centrality indices (bootstraps number = 2500) for the participants hesitating to be vaccinated (left panel) and those vaccinated (right panel).*
